# Peritumoral Cystic Meningioma With Diagnostic Challenges: Two Case Reports and a Literature Review

**DOI:** 10.7759/cureus.103583

**Published:** 2026-02-14

**Authors:** Wei-Chih Chen, Zi-Jie Lin, Lam Chee-Tat

**Affiliations:** 1 Department of Neurosurgery, Taipei Veterans General Hospital, Taipei, TWN; 2 Department of Neurosurgery, Shin Kong Wu Ho-Su Memorial Hospital, Taipei, TWN

**Keywords:** angiomatous meningioma, craniotomy for tumor, cystic neoplasm, malignant neoplasm of brain, meningioma

## Abstract

Cystic meningiomas are relatively rare and pose significant diagnostic challenges because their heterogeneous cystic architecture closely mimics gliomas or metastatic lesions. This radiological overlap creates critical diagnostic dilemmas that directly influence surgical strategy and patient counseling. We present two surgically treated cases that illustrate distinct imaging pitfalls and offer practical insights for advanced image-based differentiation. The first patient, a 45-year-old man, presented with behavioral changes and right-sided weakness; Computed tomography (CT) and magnetic resonance imaging (MRI) revealed a heterogeneous left temporal cystic mass, initially suspected to be a cystic meningioma or metastatic tumor. The second patient experienced dizziness for one day following mild trauma; The CT and MRI showed cystic lesions at the left frontal convexity. The differential diagnoses included cystic meningioma, hemangioblastoma, metastatic tumor, brain abscess, and chronic subdural hematoma. Both patients underwent frontotemporal craniotomy, and the first case revealed a cyst containing xanthochromic fluid and distinct mural nodules attached to the dura. In the second case, the cystic component contained an organized mixed hematoma, liquefied “black oil” blood, combined with granulation tissue and a solid tumor mass. Histopathology confirmed angiomatous meningioma in the first case and atypical meningioma in the second. Despite the diagnostic complexities, both patients showed favorable postoperative recovery. These cases underscore the importance of distinguishing cystic meningiomas from other intracranial pathologies.

## Introduction

Meningiomas, which arise from arachnoidal cells, constitute approximately 39% of primary intracranial tumors [1]. The classification of cystic meningioma often follows the method described by Nauta et al. [2]: Type 1, the cyst is located entirely within the tumor, in a central position; Type 2, the cyst is located at the periphery of the tumor but still contained within the tumor mass; Type 3, the cyst is situated outside the tumor, within the adjacent brain parenchyma; Type 4, the cyst is located between the tumor and the brain, often in the subarachnoid space. Rengachary simplifies these categories into two main types: intratumoral versus peritumoral cysts [3].

Cysts associated with meningiomas are uncommon, with reported incidence rates varying from 1.6% to 10% [3]. Cystic meningiomas present a significant preoperative diagnostic challenge because they can be difficult to distinguish from other cystic intracranial lesions such as gliomas, hemangioblastomas, or metastatic tumors. The diagnostic accuracy of MRI for cystic meningioma has been reported to be approximately 78% preoperatively [4]. In this study, we present two cases of cystic meningioma, each characterized by a large cyst with associated solid mass. The first case was previously presented as a E-poster at the 2024 Asian Australasian Congress of Neurological Surgeons (AACNS) Annual Scientific Meeting on November 9, 2024.

## Case presentation

Case 1

**Figure 1:**
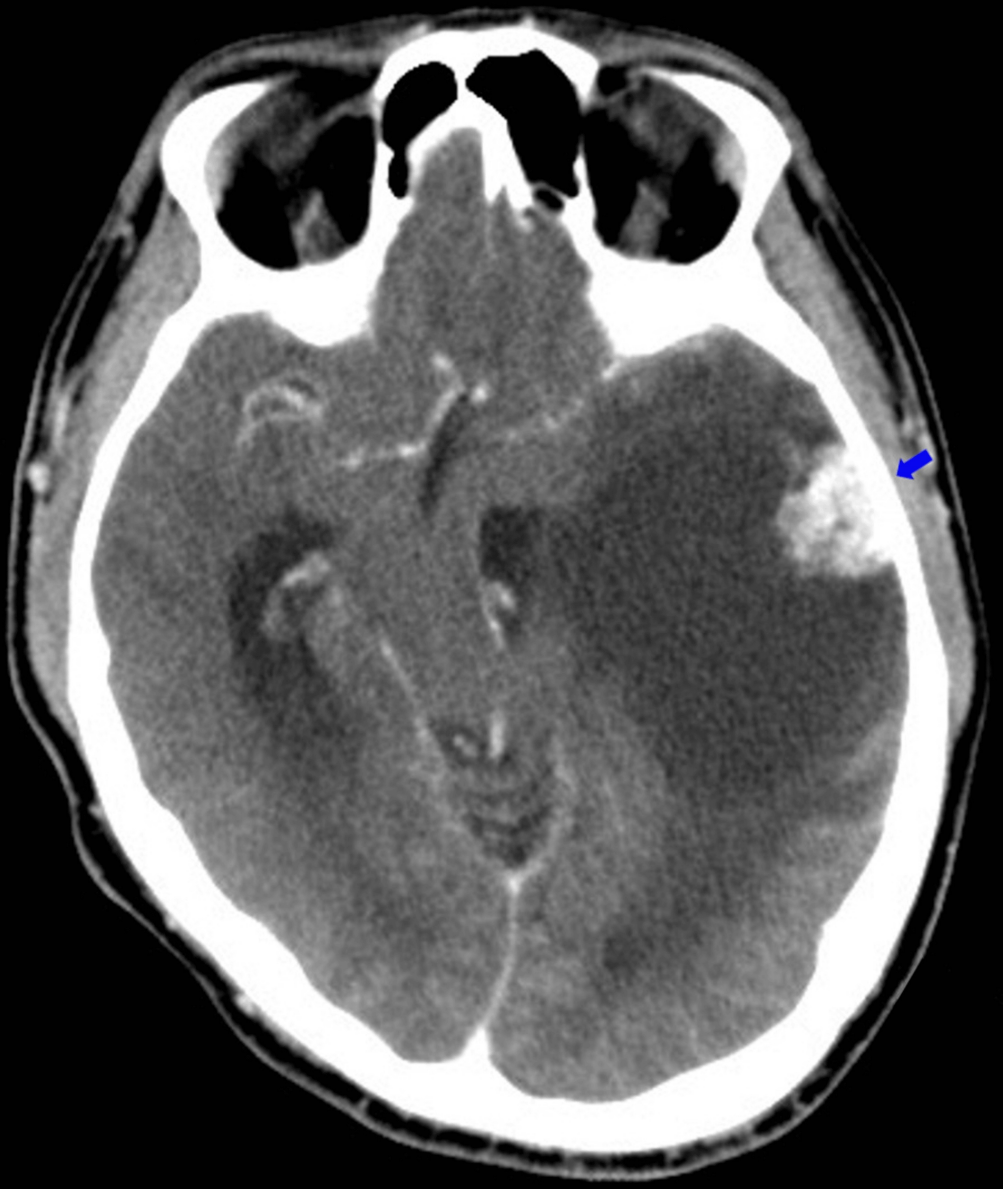
Brain CT with contrast of case 1 A tumor was identified in the left temporal lobe, with a peripherally located solid component showing contrast enhancement (blue arrow). The cystic component caused significant mass effect, resulting in midline shift to right side and uncal herniation.

**Figure 2:**
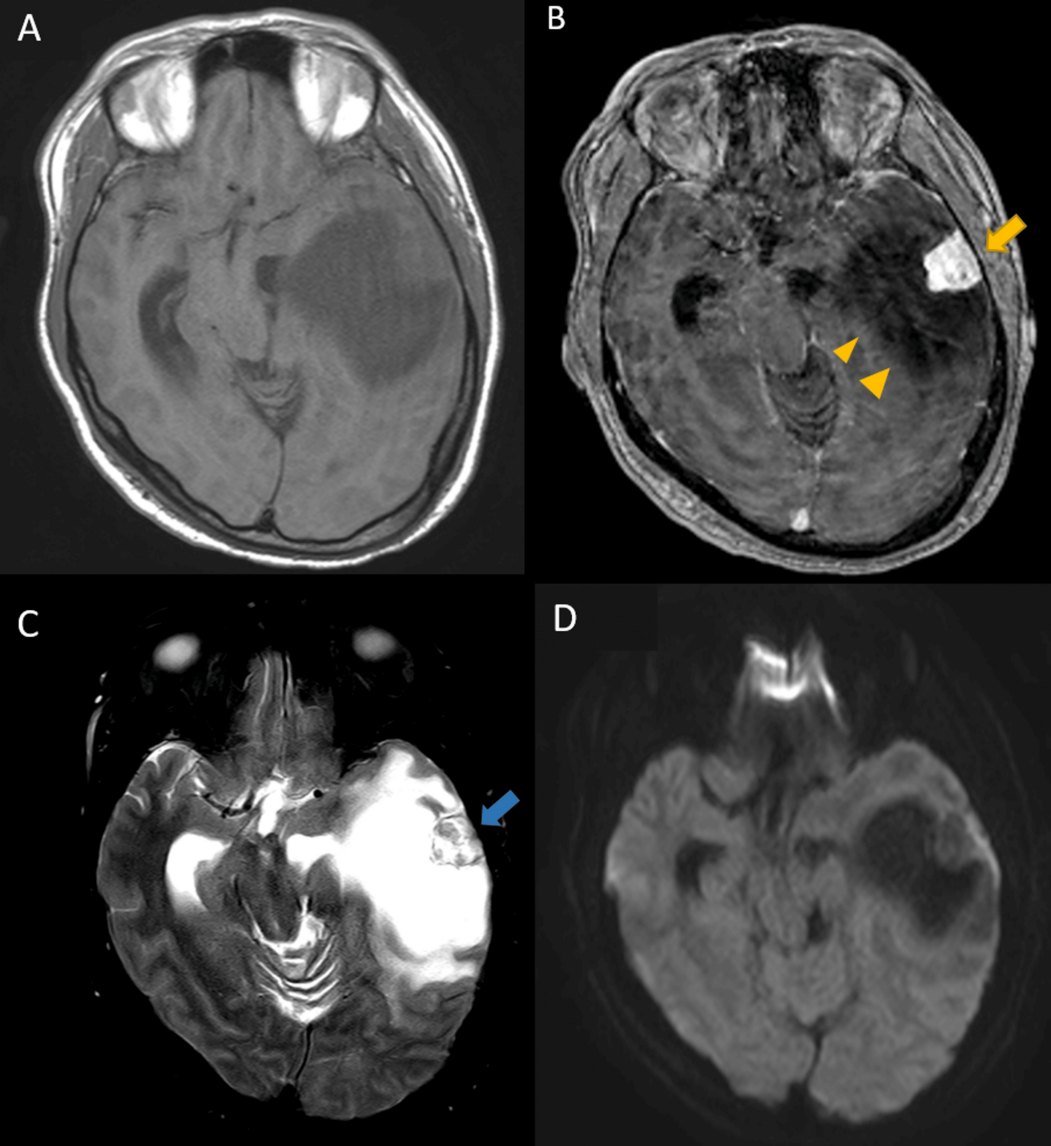
Brain MRI images of case 1 The solid part (3.1x1.9x2 cm) was isointense and the peritumoral cyst was hypointense on T1-weighted images (A). The solid part had strongly enhancing on contrast (B, yellow arrow) and heterogeneous on T2-weighted images (C, blue arrow). Peritumoral cyst (8.2x6.4x4.7 cm) was hyperintense on T2WI (C) and no contrast enhancement, including the cystic wall (B, yellow arrowhead). The tumor was showing no restriction on diffusion-weighted imaging (D). No dural tail sign was observed.

**Figure 3:**
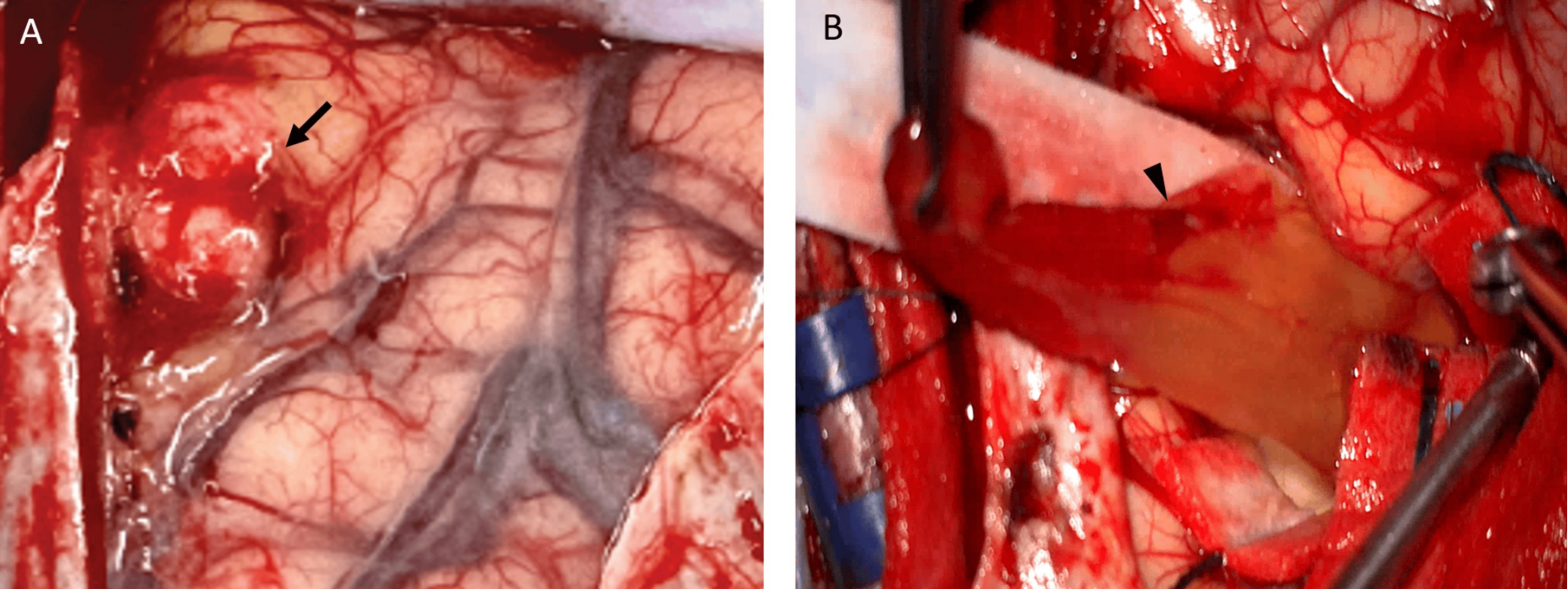
Intraoperative findings of case 1 Intraoperative findings showed a well-defined tumor (black arrow) with a soft-to-firm consistency was removed (A). Aspiration of the cystic contents yielded xanthochromic fluid, after which the cyst wall was carefully dissected from the surrounding brain parenchyma. The tumor capsule including cyst wall demonstrated clear margins without evidence of brain parenchymal invasion (B, black arrowhead).

A previously healthy 45-year-old man presented with a two-week history of atypical behavior, accompanied by headaches, dizziness, and slow, irrelevant speech. Upon neurological examination, the patient was found to have a confused conscious level with a Glasgow Coma Scale score of E4V4M6, disoriented, and mild weakness in the right limbs. The left temporal tumor was seen on a brain CT scan, which showed contrast enhancement on solid component (Figure 1). The MRI Imaging showed a lesion comprising two components: a peripherally located, strongly enhancing solid part measuring 3.1x1.9x2 cm (anteroposterior, transverse, and craniocaudal) and a large cystic part measuring 8.2x6.4x4.7 cm (anteroposterior, transverse, and craniocaudal) caused a significant mass effect (Figure 2). Cystic meningioma, metastasis, and hemangioblastoma were considered in the differential diagnosis. Cystic meningioma was deemed the most likely diagnosis based on the lesion’s location, single lesion, homogeneous enhancement, and absence of diffusion restriction on diffusion-weighted imaging, despite the lack of dural thickening. Following a frontal-temporal craniotomy, a dural attachment of the mass was identified. The cyst contained xanthochromic fluid. The tumor itself was grayish-white, soft to firm in consistency, friable, and attached to the overlying dura mater. The tumor was removed completely. Coagulative procedure was performed on the adherent dura after removal of the mass (Figure 3).

**Figure 4:**
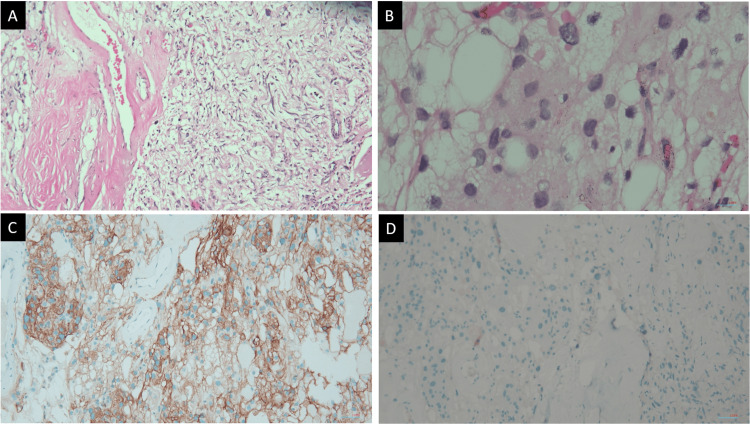
Pathohistological findings of case 1 Section shows (A) rich, thin-walled and thick-walled blood vessels and scattered meningothelial cells in the tumor; >50% of the tumor area is composed of blood vessels (H&E, x100), (B) significant nuclear pleomorphism without hypercellularity or mitotic figures (H&E, x400). (C) Vast majority of meningothelial cells show diffuse and strong membranous reactivity to SSTR2a (x100). (D) Complete absence of STA6 positivity (x100).

Histopathology was suggestive of an angiomatous meningioma with cystic changes (World Health Organization (WHO) grade 1). The tumor tissue was composed of a proliferation of vessels of varying sizes and focal meningothelial cells containing ovoid nuclei and indistinct cell border. The tumor cells showed membranous-cytoplasmic positivity for SSTR2a and were negative for STAT6 (Figure 4). No tumor cells were identified in the cyst wall.

Case 2

**Figure 5:**
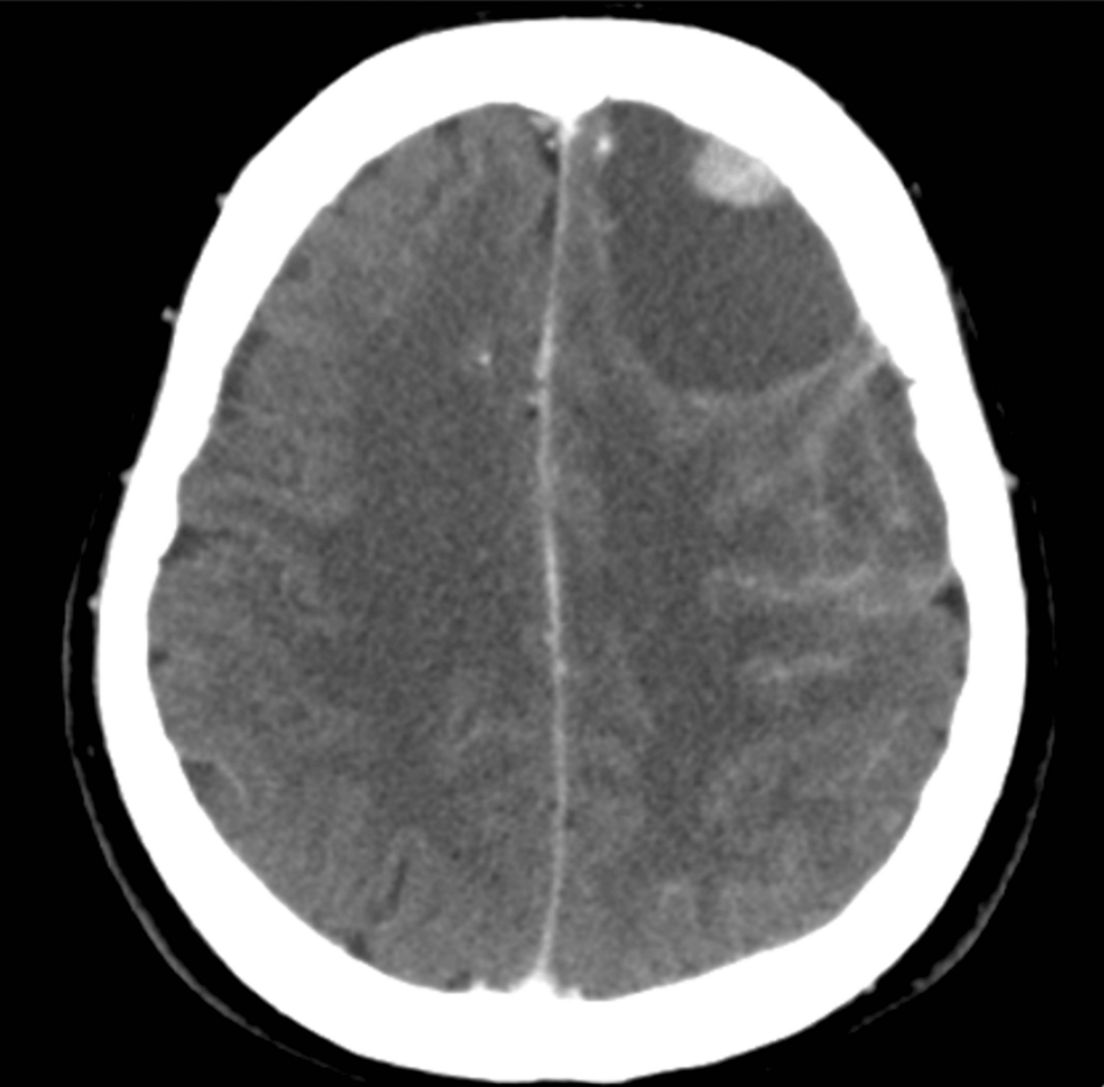
Brain CT without contrast of case 2 A heterogeneous, globular lesion in the left high frontal region. The differential diagnosis included cystic meningioma, hemangioblastoma and metastatic tumor.

**Figure 6:**
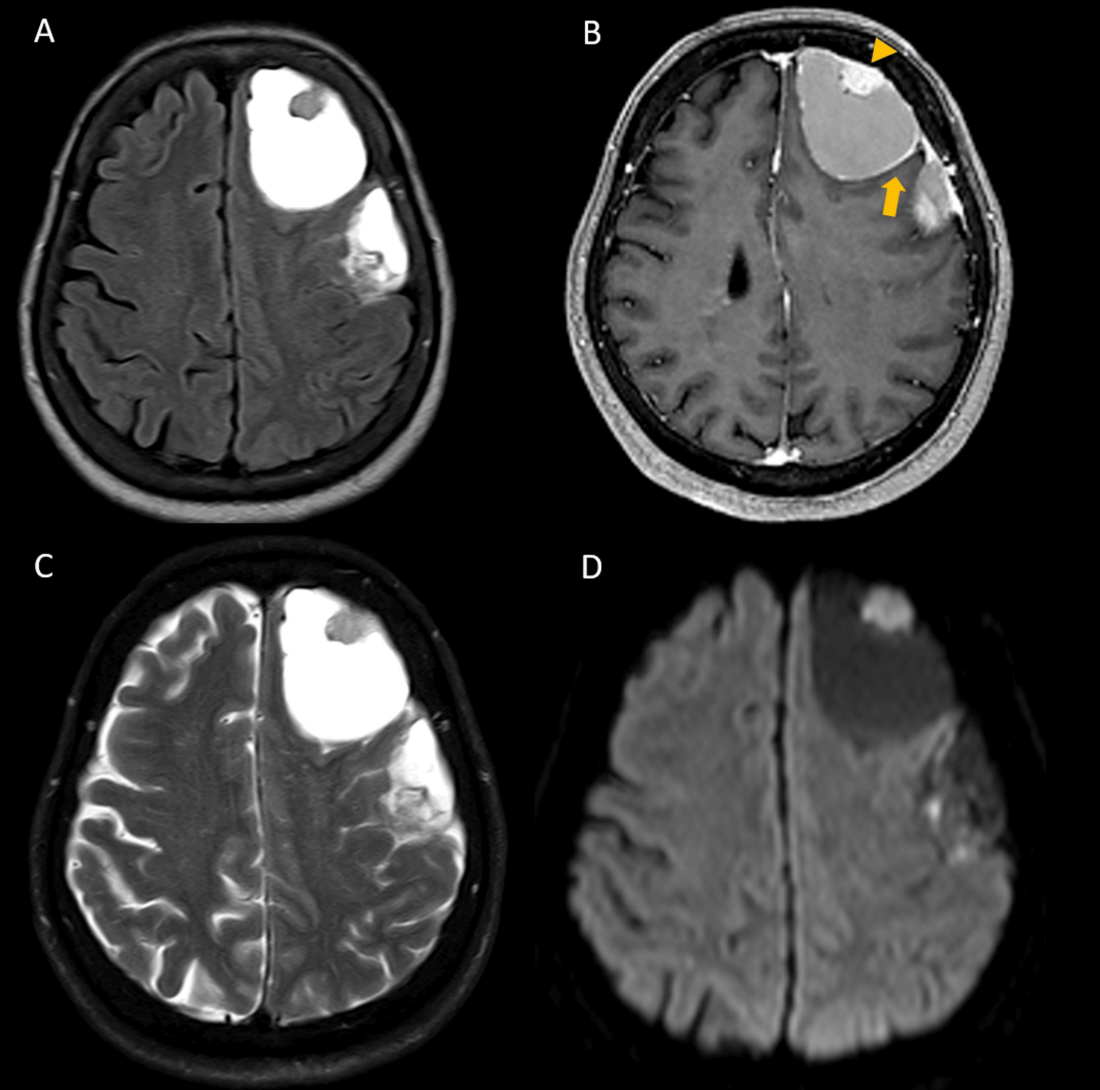
Brain MRI images of case 2 Two extra-axial cystic lesions, measuring 6.5 and 5.7 cm, respectively, with hyperintensity signal on both T1 (A) and T2-weighted images (C). Focal contrast enhancement (yellow arrowhead) was noted at the mural nodules and mild enhancement at cyst walls (B, yellow arrow). Corresponding hyperintense signal changes on diffusion-weighted imaging (DWI) were observed at these enhancing sites (D).

**Figure 7:**
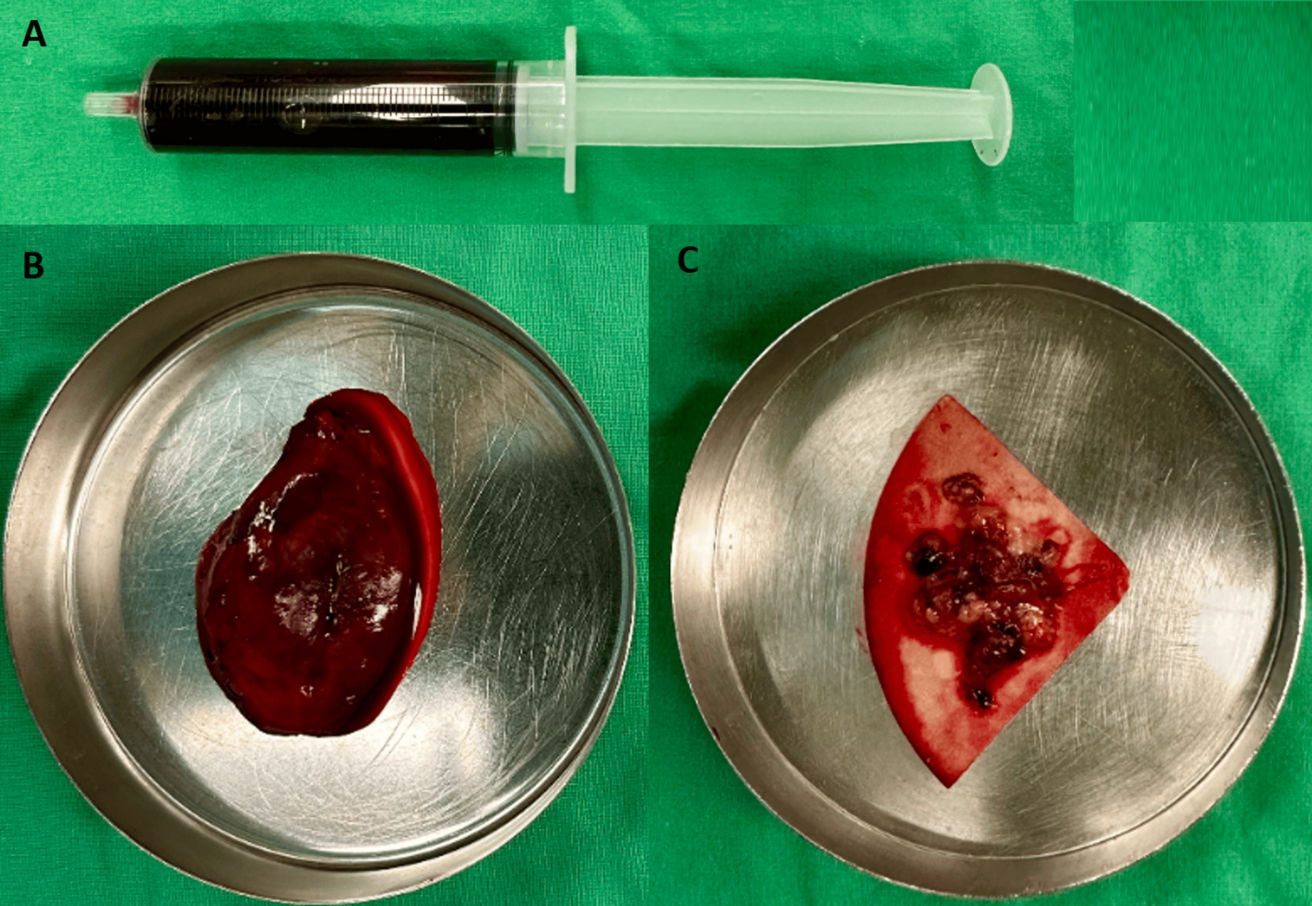
Operative findings of case 2 The tumor cyst contained liquefied material that was aspirated using a syringe (A), along with an organized mixed hematoma (B). The solid tumor component appeared as granulation-like tissue with yellowish-gray coloration (C).

**Figure 8:**
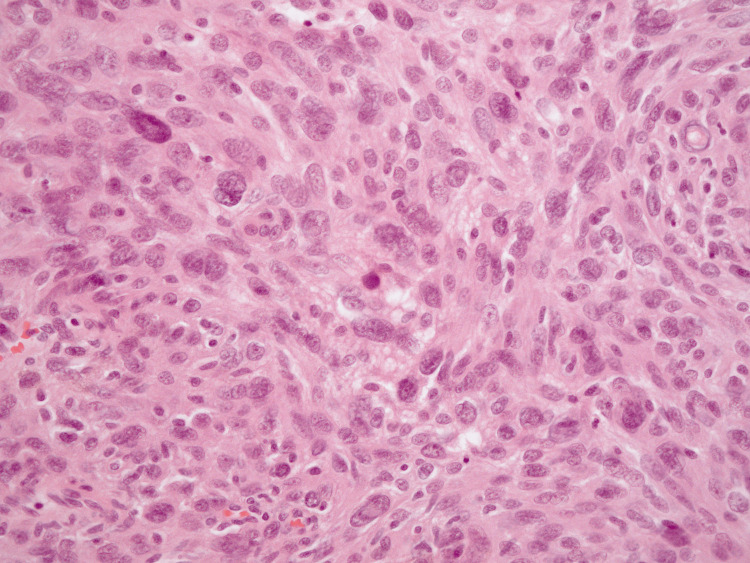
Histopathological diagnosis of case 2 (Hematoxylin and eosin (H&E), ×400). The specimen demonstrate a proliferation of spindle-shaped meningothelial cells arranged in whorled patterns and sheet-like formations. Nuclear atypia, prominent nucleoli, and increased cellularity are evident. The mitotic index is 5 per 10 high-power fields. These features are consistent with an atypical meningioma (WHO Grade 2).

A 66-year-old woman with a history of hypertension, well controlled with medication, presented with the sudden onset of progressively worsening dizziness lasting one day following a mild traumatic event. On arrival at the emergency department, she was amnestic and disoriented, with a Glasgow Coma Scale score of E4V4M6. Mild fever was also noted. She had no focal neurological deficits, nausea, or vomiting. Laboratory studies revealed no significant abnormalities. Brain CT and MRI revealed extra-axial cystic lesions at the left frontal convexity, measuring approximately 6.5 cm and 5.7 cm, respectively (Figures 5, 6). Frontal cystic lesions were identified on imaging. The differential diagnoses included cystic meningioma, hemangioblastoma, metastatic tumor, brain abscess, and chronic subdural hematoma. Brain abscess and chronic subdural hematoma were considered less likely because there was no diffusion restriction within the cystic component on diffusion-weighted imaging, and a solid enhancing component was present. Hemangioblastoma and a hemorrhagic tumor were considered more likely due to the cystic component associated with hemosiderin-related signal changes, which appeared hyperintense on both T1- and T2-weighted images. Given the presence of two adjacent lesions, hemorrhagic metastatic tumors were initially favored. The solid components demonstrated contrast enhancement, and the presence of diffusion restriction within the enhancing portions further supported this consideration. Due to the above findings, a left frontal craniotomy was performed. Intraoperatively, the cyst was found to contain an organized mixed hematoma, along with liquefied “black oil” blood, granulation tissue and a solid tumor component. Complete resection all of the lesions was achieved (Figure 7). Histopathological examination revealed a proliferation of meningothelial cells with nuclear atypia. The mitotic index is 5 per 10 high-power fields (HPFs). Atypical meningioma (WHO Grade 2) was diagnosed (Figure 8).

## Discussion

Several theories have been proposed for cyst development. Intratumoral cysts are generally linked to degenerative changes within the tumor, such as microcystic degeneration, ischemic necrosis, or intratumoral hemorrhage. Rapidly growing tumors show a higher tendency toward necrosis [3]. Peritumoral cysts (Nauta types 3 and 4), which may be caused by peritumoral edema, widening of the subarachnoid space, demyelination, or peritumoral hemorrhage, represent a distinct process from intratumoral cysts. Peritumoral cysts are typically large, unilocular, and filled with xanthochromic, protein-rich fluid. The proposed mechanisms include reactive gliosis and breakdown of the brain-tumor interface (Nauta type 3), which means chronic peritumoral edema may evolve into cyst formation. Another possibility is the CSF entrapment mechanisms (Nauta type 4), in which mechanical obstruction of the tumor may trap cerebrospinal fluid between the tumor capsule and the arachnoid plane. The expansion of the subarachnoid space leads to cyst formation [3,5]. In our study, case 1 was classified as Nauta type 3, in which the cyst wall contained no tumor cells. The mechanism of cyst formation in this type is thought to be related to peritumoral edema and reactive gliosis. Case 2 was classified as Nauta type 4, in which both the cyst and the tumor were extra-axial and located outside the subarachnoid space. The clinical, radiological, and pathological characteristics of both cases are summarized in Table 1.

**Table 1:** Comparative Summary of Clinical Cases A summary of basic data, symptoms, location, Nauta classification, radiological and pathological characteristics of the two patients with cystic meningioma presented in this study.

	Case 1	Case 2
Age(years)	45	66
Sex	Male	Female
Presenting symptoms	Behavioral changes; right-sided weakness	Dizziness; disoriented
Tumor location	Left temporal	Left frontotemporal
Nauta classification	Type 3	Type 4
T1-weighted (solid component)	Isointense	Isointense
T1-weighted (cystic component)	Hypointense	Hyperintense
T2-weighted (solid component)	Heterogeneous	Isointense
T2-weighted (cystic component)	Hyperintense	Hyperintense
Enhancement	Enhancement of solid component; no cyst wall enhancement	Enhancement of solid component; mild cyst wall enhancement
Diffusion-weighted imaging (solid component)	No restriction	Diffusion restriction present
Diffusion-weighted imaging (cystic component)	No restriction	No restriction
Histopathology	Angiomatous meningioma	Atypical meningioma

Meningiomas are classified according to the World Health Organization (WHO) 2021 grading system and divided into three grades: grade 1 meningiomas are considered benign and are characterized by a low mitotic rate (less than four mitoses per 10 HPFs) and the absence of brain invasion. Grade 2 (atypical) meningiomas have either a mitotic rate of 4-19/10 HPF, brain invasion, or at least three of five specific histologic features (necrosis; increased cellularity; small cell change; prominent nucleoli; patternless or sheet-like growth). Grade 3 meningiomas have either a mitotic rate >20/10 HPF or an overtly malignant appearance. The distribution of documented meningioma cases are 80% grade 1, 18% grade 2, and 2% grade 3 [6]. In a small study evaluated 38 cases of cystic meningioma, meningothelial (36.8%), transitional (15.8%), fibrous (26.3%), atypical (21.1%) [7]. In a meta-analysis evaluating CT and MRI features for differentiating high-grade (WHO grades 2 and 3) from low-grade (WHO grade 1) meningiomas, cystic changes within the tumor were significantly associated with tumor grade and demonstrated the highest specificity (93.4%) among other imaging features, including irregular tumor-brain interface, mass effect, heterogeneous tumor enhancement, focal edema, and so on [8]. Meningiomas with hemorrhage is an uncommon but well-documented phenomenon, with an estimated incidence ranging around 1.3%-2.4% of all intracranial meningiomas [9]. The most common location was the convexity (around 50%), followed by falcine, parasagittal, sphenoid wing, and trigone [9,10]. The proposed mechanisms include abnormal tumor angiogenesis, fragile or dysplastic intratumoral vessels, increased microvascular density and endothelial proliferation. Furthermore, certain histological subtypes, including meningotheliomatous, fibrous, angiomatous, transitional, and malignant, have been recurrently observed in hemorrhagic meningioma cases. But statistical significance was only observed for fibrous meningiomas. Angiomatous meningiomas and aggressive (grades 2 and 3) are not shown to be associated with an increased risk of hemorrhage [10,11]. However, hemorrhagic presentation of meningiomas is linked to poorer clinical outcomes. In the modern era (post-1980), the reported mortality rate for hemorrhagic meningiomas is approximately 7.5%, which is twice that observed in non-hemorrhagic meningiomas. Postoperative outcomes largely depend on the patients’ preoperative neurological condition, as 64% of patients who died were comatose prior to surgery [11].

Angiomatous meningioma is a rare histological subtype of benign meningioma, classified as a WHO grade I tumor, and accounts for approximately 2.1% of all meningiomas. It presents as cystic meningioma in 14.8% of cases and has a silent tumoral growth and a low proliferation and infiltration rate. Despite being benign nature and comparatively small in size, they look aggressive on radiology images like massive peritumoral edema and intense contrast enhancement [12,13].

The preoperative diagnosis of cystic meningiomas remains difficult, as their radiological appearance may lead to diagnostic confusion with metastatic tumors or gliomas, particularly when associated with disorganized morphological changes and prominent vasogenic edema [4,14]. Metastatic tumors may be seen at multiple locations. High-grade gliomas may demonstrate necrosis, heterogeneous enhancement, and diffusion restriction on diffusion-weighted images (DWI). Another differential diagnosis for cystic meningioma is hemangioblastoma; although hemangioblastomas most commonly arise in the cerebellum, their imaging characteristics can resemble those of cystic meningiomas when a cystic component is present. Radiologically, hemangioblastomas classically present as a cyst with an enhancing mural nodule. On CT imaging, the solid component is typically isodense to brain parenchyma, and calcification is usually absent. On MRI, the mural nodule is generally T1 hypointense to isointense and T2 hyperintense, often demonstrating prominent flow voids that reflect the tumor’s hypervascular nature. These lesions are frequently seen abutting the pia surface rather than the dura. Importantly, when a cystic component is present, the cyst wall in hemangioblastoma rarely enhances [15].

## Conclusions

Cystic meningiomas exhibit heterogeneous radiological appearances, variable cyst locations, and focal edema, which may result in diagnostic confusion with other cystic intracranial lesions such as gliomas, metastatic tumors, or hemangioblastomas. As demonstrated in our two cases, cystic meningiomas can present with markedly different pathological grades and underlying mechanisms, ranging from a benign angiomatous meningioma to an atypical meningioma complicated by hemorrhage and cystic degeneration, supporting the association between cystic change and more aggressive or higher-grade pathology. Furthermore, tumor-related hemorrhage may be associated with increased mortality and poorer clinical outcomes. Awareness of the diverse presentations and pathophysiological mechanisms of cystic meningiomas may help neurosurgeons and clinicians reduce misdiagnosis, optimize surgical strategies, and improve patient outcomes.
